# Zumba^®^, Fat Mass and Maximum Oxygen Consumption: A Systematic Review and Meta-Analysis

**DOI:** 10.3390/ijerph18010105

**Published:** 2020-12-25

**Authors:** Manuel Chavarrias, Santos Villafaina, Ana Myriam Lavín-Pérez, Jorge Carlos-Vivas, Eugenio Merellano-Navarro, Jorge Pérez-Gómez

**Affiliations:** 1 Health, Economy, Motricity and Education Research Group, Faculty of Sport Sciences, University of Extremadura, 10003 Cáceres, Spain; manuelchavarrias@gmail.com (M.C.); jorge.carlosvivas@gmail.com (J.C.-V.); jorgepg100@gmail.com (J.P.-G.); 2 Faculty of Sport Science, University of Extremadura, 10003 Cáceres, Spain; 3 Centre for Sport Studies, Rey Juan Carlos University, 28943 Fuenlabrada, Spain; am.lavin.2018@alumnos.urjc.es; 4 Grupo de Investigacion EFISAL, Universidad Autónoma de Chile, 3460000 Talca, Chile; emerellano@gmail.com

**Keywords:** body mass, fitness, obesity, overweight, VO_2max_, Zumba^®^

## Abstract

Background and objectives: Obesity or overweight is associated with many health risk factors and preventable mortality. Even people with normal weight and without history of obesity or overweight should avoid weight gain to reduce health risks factors. In this regard Latin aerobic dances involved in Zumba^®^ practice make this modality motivating for people. Apart from weight loss and VO2_peak_ benefits, Zumba practice is also interesting by the increase in adherence which can also avoid weight regain. The aim was to systematically review the scientific literature about the effects of any randomized intervention of Zumba^®^ practice on total fat mass (%) and maximum oxygen consumption (VO_2peak_), besides establishing directions for the clinical practice. Evidence acquisition: Two systematic searches were conducted in two electronic databases following the PRISMA guidelines. The eligibility criteria were (a) outcomes: body mass or VO_2peak_ data including mean and standard deviation (SD) before and after Zumba^®^ intervention, (b) study design: randomized controlled trial (RCT) and (c) language: English. GRADE guidelines were used to assess the quality of evidence. A meta-analysis was performed to determine mean differences. Nine and four studies were selected for fat mass percentage and VO_2peak_ in the systematic review, respectively. However, only eight studies for fat mass percentage and three for VO_2peak_ could be included in the meta-analysis. Evidence synthesis: The overall standardized mean difference for fat mass was −0.25 with a 95% CI from −0.67 to 0.16 with a p-value of 0.69, with large heterogeneity. On the other hand, the overall effect size for VO_2peak_ was 0.53 (95% CI from 0.04 to 1.02 with a p-value of 0.03) with large heterogeneity. Conclusions: Based on the evidence, we cannot conclude that Zumba^®^ is effective at reducing body mass but it may improve VO_2peak_. However, the limited number of studies that met the inclusion criteria makes it too early to reach a definite conclusion, so more research is needed.

## 1. Introduction

Obesity (body fat mass percentage greater than 25% or 35% in men or women respectively) [1,2,3] is associated with many health risk factors [4,5] and preventable mortality [6,7]. Even people with normal fat mass and without history of obesity or overweight should avoid weight gain to reduce health risks factors [8]. Higher body fat percentage can lead to a high risk of for cardiovascular diseases, coronary events [9,10], and all-cause mortality [11,12]. However, weight management is complex, and most people do not sustain weight loss over time [13]. Therefore, it is relevant not only to fat mass or weight but also to sustained weight loss (3–5%) because it may lead to decreases in cardiovascular risk factors [8]. Thus, it is essential to find training intervention for weight management.

Poor cardiorespiratory fitness is also associated with chronic disease and premature mortality [14,15]. The maximum oxygen uptake (VO_2peak_) is the main and gold standard measure for cardiorespiratory fitness [16]. Aerobic training can decrease chronic disease by increasing VO_2peak_ through many adaptations like improvements in cardiac size, cardiac output, stroke volume or mitochondrial function and number [14,15]. In order to increase the quality of life as well as to avoid health problems, many physical activities guidelines have been appeared around the world [17,18]. However, few peoples follow the guidelines, leading to sedentary behaviors which impair the cardiorespiratory fitness. This could be due to a lack of motivation to enroll in exercise interventions.

Lifestyle interventions can be a preventive strategy for illness without adverse [19]. In this regard, Zumba^®^ is one of the most popular exercise programs in recent years that involves many health benefits [20], including parameters related to the quality of life (such as, physical self-perception and psychological well-being), anthropometric, body composition, blood pressure, and physical fitness [20,21].

Latin aerobic dances involved in Zumba^®^ practice make this modality motivating for people. Apart from weight loss and VO_2peak_ benefits [20], Zumba^®^ practice is also interesting by the increase in adherence a key variable in the weight loss interventions [22]. Therefore, the purpose of this study was to conduct a systematic review of the scientific literature to explore the effects of Zumba^®^ practice on fat mass percentage and VO_2peak_, in order to provide clinical practice recommendations. Furthermore, two meta-analyses were aimed to determine the effect sizes of Zumba^®^ interventions on the reduction of fat mass and the increase of VO_2peak_.

## 2. Materials and Methods

The present systematic review was conducted following the statements and guidelines included in the Preferred Reporting Items for Systematic Reviews and Meta-Analyses Guidelines (PRISMA), for search procedures, study selection and data collection and analysis [23].

### 2.1. Literature Search

The literature search was conducted in two different electronic databases: PubMed (Medline) and Web of Science (including Current contents connect, Derwent innovations index, Korean journal database, Medline, Russian science citation index, Scielo citation index). Two different searches were conducted in October 2020. Boolean search phrases in all of the mentioned databases were: (1) (“zumba”) AND (“waist circumference” OR “waist-hip” OR “fat” OR “weight” OR “BMI” OR “body composition” OR “body mass”); and (2) (“zumba”) AND (“vo2” OR “oxygen” OR “VO2max”). The exact search strings for each databases and variables (total fat mass or VO2_peak_) are shown in Supplementary File 1.

### 2.2. Study Selection and Eligibility Criteria

Two independent evaluators selected the potentially eligible articles from the databases (MC and JPG). There were no disagreements between them. To be included into this review, studies need to meet the following eligibility criteria: (a) studies need to report fat mass or VO_2peak_ data, with means ± standard deviation (SD) before and after the Zumba^®^ training intervention, (b) studies should be randomized controlled trial (RCT) and (c) manuscripts must be written in English language.

### 2.3. Quality of Evidence and Risk of Bias

The Grading of Recommendations, Assessment, Development and Evaluation system (GRADE) [24] was used to evaluate the quality of evidence. According to the GRADE system, the present article was initially classified as a high evidence because all studies included are RCTs, but the evidence dropped twice due to imprecision (small sample size and 95% CI of the mean difference including the value “0”). Therefore, the final quality of the evidence was low, which means that the confidence in the effect estimate is limited.

Additionally, the Cochrane Collaboration’s tool for assessing risk of bias was applied to evaluate the risk of bias [25]. This instrument classified the selection, performance, detection, attrition and reporting bias as low, high or unclear risk of bias.

### 2.4. Data Collection

Data extraction from all the studies was independently conducted by two different authors (S.V. and J.C.-V.). The obtained information included number and type of participants, interventions characteristics, comparisons, outcomes, and study design (PICOS), following the recommendations collected into the PRISMA statement. Table 1 shows the characteristics of participants regarding age, sex, sample size and distribution by groups. Table 2 presents the characteristics of interventions and the comparison groups, including number of sessions, training frequency (days per week) and total duration of every study. Tables 3 and 4 display results for the different main outcomes (fat mass percentage and VO2_peak_). Study design was not included in any table because all studies were RCT.

### 2.5. Statistical Analysis

The main outcomes of this meta-analysis were total fat mass percentage (%) and VO_2peak_. Tables 3 and 4 display the results of all studies on these variables. Treatment effects were calculated as the difference between the change of Zumba^®^ group and the change of the control group (inactive and active control groups). Effect sizes were calculated for each study using the reported sample sizes, means and standard deviations (SDs) before and after the treatment, or with its calculation through the use of standard error. Heterogeneity was evaluated by calculating the following statistics: (a) Tau^2^, (b) Chi^2^, and (c) I^2^. The most common classification of I^2^ consider values lower than 25% as small heterogeneity, values between 25 and 50% as medium, and higher than 50% were considered large.

All analyses were conducted using the Review Manager Software (RevMan, version 5.3, Cochrane Collaboration, Oxford, UK) for Windows. The Standardized Mean Difference (SMD) was calculated and a random model was used.

## 3. Results

### 3.1. Study Selection

**Figure 1:**
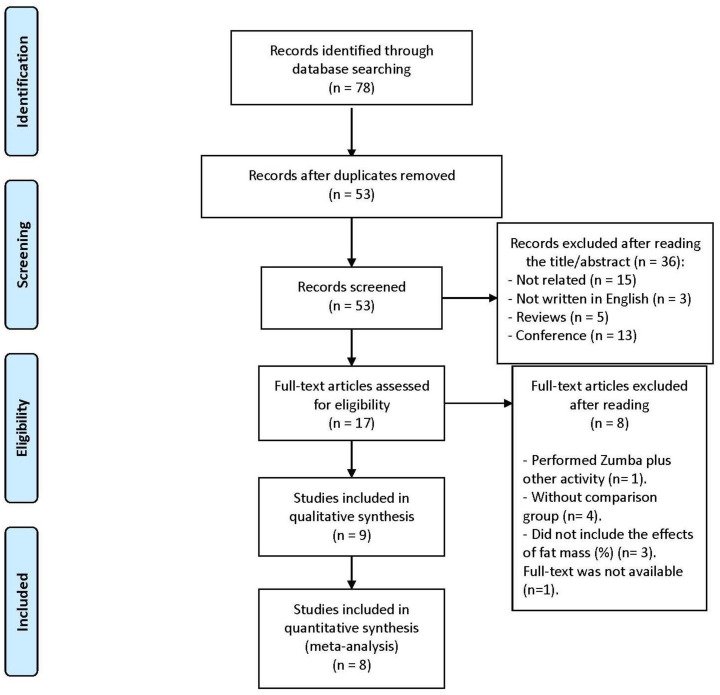
Study selection flow chart, about total fat mass (%), according with PRISMA statements.

**Figure 2:**
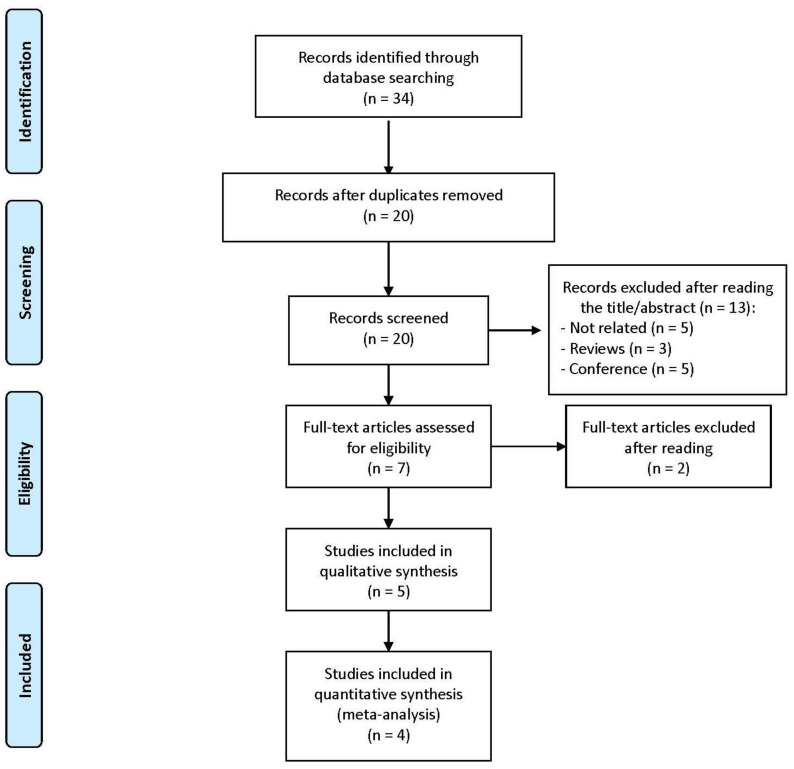
Flow chart for selection of studies, about VO_2peak_, according with PRISMA statements.

Figure 1 and Figure 2 shows the PRISMA flow diagrams for fat mass and VO_2peak_ searches, respectively. As it is displays in Figure 1, a total of 78 records were initially identified, 25 of which were removed because they were duplicates. Of the remaining 45 articles, 15 were excluded because they were not related to the main topic of this review, three studies were not written in English, five were reviews and 13 were conference papers. After reading the remaining 17 studies, nine of them were excluded because they did not meet the eligibility criteria. Finally, eight articles were included in the systematic review [21,26,27,28,29,30,31,32,33], and eight articles were suitable for the meta-analysis [21,26,27,29,30,31,32,33]. However, Barene et al. published two articles [27,28] that were part of the same trial (ISRCTN61986892), thus only one of the two articles was included in the meta-analysis to avoid methodological problems in the meta-analysis. In order to increase the homogeneity of studies, the 12-week study [27] was selected instead of the 40-week study. Meta-analysis results were very similar including any of those two studies, not achieving significant effects in any of them.

As it is shown in Figure 2, a total of 34 studies were initially identified, 14 of them were removed because they were duplicates. Of the remaining 20 studies, five were excluded because they were not related to the main topic, three were reviews and five were conference papers. After reading the remaining seven studies, three did not meet the inclusion criteria and were excluded. Finally, five studies were suitable for inclusion in the systematic review [21,26,27,28,34] and four in the meta-analysis [21,26,34], but again, only the 12-week study by Barene, Krustrup, Jackman, Brekke and Holtermann [27] was included.

### 3.2. Quality of Evidence and Risk of Bias

According to the GRADE system, the present article was initially classified as a high evidence because all studies included are RCT, but the evidence dropped two levels due to imprecision (small sample size and 95% CI of the mean difference including the value “0”). Thus, the final quality of the evidence was low, which means that the confidence in the estimate effect is limited.

**Figure 3:**
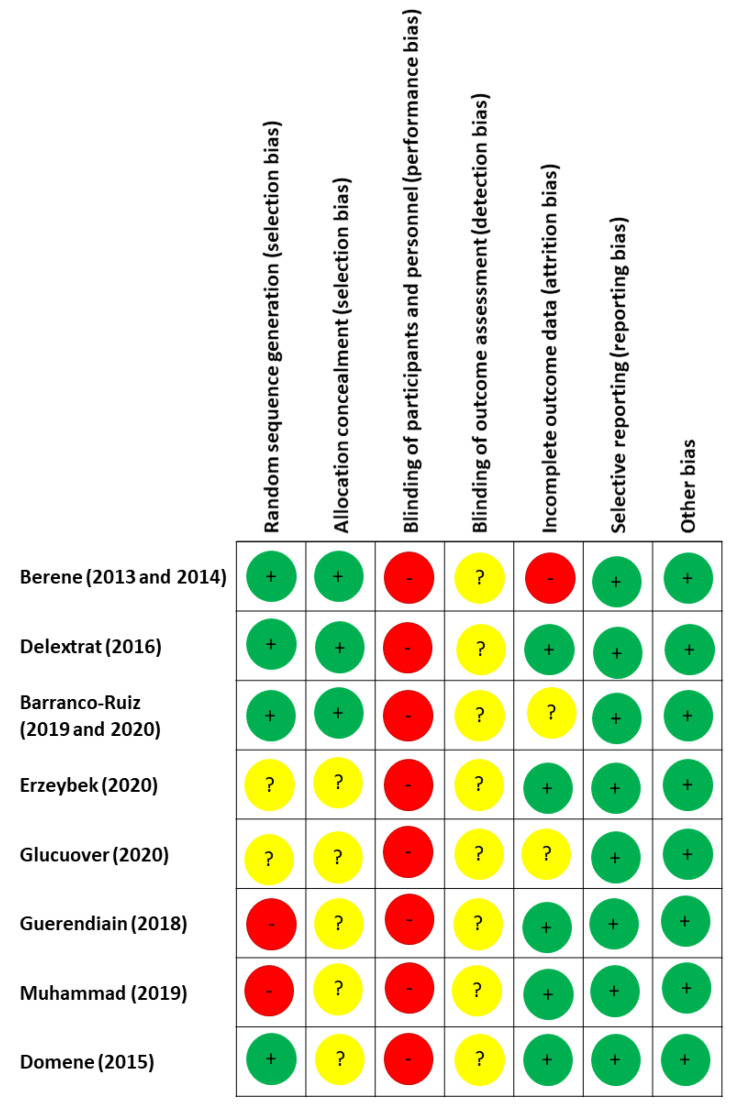
The Cochrane Collaboration’s tool for assessing risk of bias.

The Cochrane Collaboration’s tool for assessing risk of bias was employed in the present study (see Figure 3). Seven sources of bias were assessed as low (green), high (red) and unclear (yellow). The poorer scores were obtained in blinding participants and personnel (any study reported it), blinding of outcomes assessment (unclear in all the studies), allocation concealment (in five article was unclear) and random sequence generation (two articles did not report and in other two is unclear).

### 3.3. Study Characteristics

Table 1 summarizes the characteristics of the different studies included. A total sample of 373 participants was included in the systematic review (excluding those participants who were duplicated from articles, as happens with the manuscripts by Barene, Krustrup, Brekke and Holtermann [28] and Barranco-Ruiz and Villa-González [34]. Of these, 196 belonged to the Zumba group and 177 were the control group. The age ranged from 18 to 47.4 years old. Most participants were females.

### 3.4. Interventions

The characteristics of the Zumba^®^ interventions and control groups are displayed in Table 2. The weekly frequency of sessions varied from 1 to 3. The total duration of the interventions ranged from 8 to 40 weeks, but the duration of the interventions included in the meta-analysis ranged from 8 to 16 weeks. Zumba^®^ sessions lasted between 45 min to one hour. The comparison group was active or passive depending on the article. Seven articles presented a passive control group, where participants had to follow their normal daily activities. Nevertheless, three articles presented an active control group with different activities such as oscillation training, Zumba^®^ training plus body weight and an educational program.

### 3.5. Outcome Measures

This systematic review included manuscripts focused on total fat mass (%) and VO_2peak_. The baseline weight values ranged from 23.5 to 36.3% total fat mass (%) and the VO_2peak_ from 28 to 38.3 mL kg^−1^ min^−1^. According to the Acsm [35] guideline fat mass (%) corresponds to poor/very poor and VO2peak corresponds to fair/excellent depending on the study. Four studies reported significant reductions in total fat mass (%) compared to the control group (see Table 3). The most relevant changes were obtained in the Barranco-Ruiz (2019) and Guerendiain et al., (2019) studies with significant decreases of 2.9% and 3.3% respectively. Total fat mass (%) was calculated using a DXA scan in two articles [27,28], using a multifrequency bioelectrical impedance analyzer in five article [21,26,30,31,32] and two articles performed anthropometric evaluation following the International Society for the Advancement of Kinanthropometry (ISAK) procedures [29,33].

Regarding VO_2peak_, three studies found significant improvement intragroup, and five studies observed significant improvement in comparison with the control group (see Table 4). The most relevant changes were found in the study of Domene et al., (2016) and Barranco-Ruiz and Villa-González (2020) with an increase of 3.1 and 1.3 mL kg^−1^ min^−1^, respectively. Three studies calculated VO2_peak_ through an incremental test on treadmill or bycicle [26,27,28], another study used a graded treadmill exercise test [21] and only one predicted it from a 2-km walking test [33].

**Figure 4:**
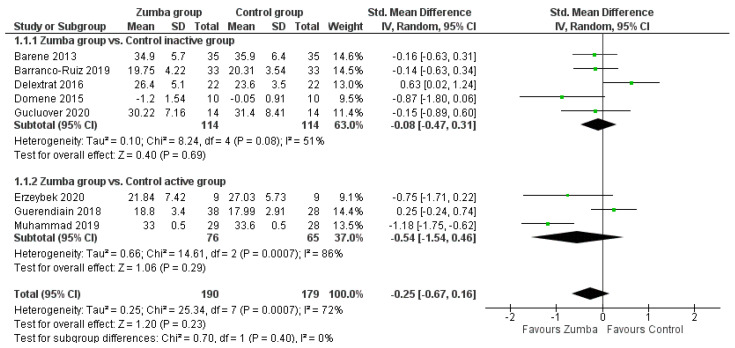
Effects of Zumba^®^ on total fat mass (%).

The standardized mean difference for the total fat mass (%) difference between control and Zumba^®^ was −0.25 with a 95% confidence interval from −0.67 to 0.16 and a *p*-value of 0.23. The heterogeneity level was high according to the I^2^ = 72%. In this meta-analysis were considering both active or passive control groups. Thus, two sub meta-analyses were created to divide between active or passive control groups. Regarding, the subgroup analyses performed showed p-values of 0.69 and 0.29 when contrasting the effects of Zumba^®^ to an inactive control group (SMD of −0.08 and a 95% CI from −0.47 to 0.31) and an active control group (SMD of −0.54 and a 95% CI from −1.54 to 0.46), respectively (see Figure 4).

**Figure 5:**
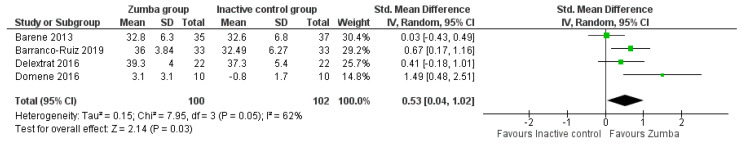
Effects of Zumba^®^ on VO_2peak_.

On the other hand, the standardized mean difference for VO2_peak_ was 0.53 with a 95% confidence interval from 0.04 to 1.02 and a p-value 0.03. The heterogeneity level was large as the I^2^ was 62% (see Figure 5). In this meta-analysis the control group consisted on a passive group, where participant did not conduct physical activity intervention or followed with their normal daily life activities.

## 4. Discussion

This study aimed to review the scientific literature to collect those studies that have explored the effects of Zumba^®^ practice on total fat mass (%) and VO_2peak_ and performed two meta-analyses (one for fat mass and another for VO_2peak_) to determine the effect sizes of Zumba^®^ on reducing fat mass and increasing VO_2peak_. The results of these meta-analyses showed that Zumba^®^ practice could be an effective tool to enhance the VO_2peak_ but an ineffective strategy for losing total fat mass (%). In this regard, one possible reason why these changes were small in fat mass is that the initial total fat mass of participants was relatively low, giving more limited opportunity for weight loss (see Table 3). Nevertheless, five of the nine selected studies showed a reduction in total fat mass (%) after the Zumba^®^ intervention when compared with a control group. The highest reduction in total fat mass (%) (3.37 of change) were observed when combining Zumba^®^ workouts plus body weight. Regarding the improvements of VO_2peak_, it would mean that enzymatic activity of mitochondria [36], and, therefore, the oxidation of fatty acids during exercise would be increased [37,38,39]. Thus, probably the participants were not able to reduce the total fat mass (%) because of their dietary intake, but certainly not because of the Zumba^®^ intervention. Moreover, since the metabolism lower with age [40,41], could be that the age of the participants (which varied in a wide range) could have an impact on this variable. Therefore, maybe, in order to be more effective, the Zumba^®^ training should be combined with other training modalities such as strength training. Importantly the vast majority of the sample was comprised by women. Due to differences in VO_2peak_ and fat mass between males and females, further studies, including both males and females’ participants, are needed to consolidate the results of this systematic review.

Zumba^®^ training could improve cardiorespiratory outcomes such as VO_2peak_ (*p*-value = 0.03). However, additional studies will elucidate if Zumba^®^ practice can be a useful activity to improve the cardiorespiratory fitness. All of the individual studies included here, showed that Zumba^®^ practice significantly improves VO_2peak_ [21,26,27,28,34] (see Table 3). However, due to the limited number of these studies and their small sample sizes, this meta-analysis could not directly identify improvements in VO_2peak_ resulting from Zumba^®^ training. Thus, in order to increase the knowledge about Zumba benefits, future studies should compare Zumba^®^ with other disciplines (spinning, body pump …), study the effects of Zumba^®^ in special populations or the injuries associated with this modality. This would be relevant since Zumba^®^ workout is performed by over 12 million people worldwide [42]. Furthermore, this future intervention should take in account both the perceived barriers and the facilitator of the dance intervention [43].

It is important that motivating aerobic physical activities like Zumba^®^ have a positive effect on cardiovascular fitness. This would allow that participants who reached the recommended physical activities levels [44] to obtain health benefits practicing an entertaining activity. In this regard, adherence is maintained only when participation is perceived as enjoyable [45]. This is relevant since latin dance and latin-themed aerobic dance, like Zumba^®^, address the element of the self-determination theory [46]. This theory supports that physical activity can be an inherently rewarding activity that contributes to happiness and subjective vitality. Intrinsic motivation (engage in an activity because of the inherent pleasure) and extrinsic motivation (engage in an activity to obtain some separable outcomes) are the most basic principles in this theory [25]. In this regard, we hypothesized that Zumba^®^, which combines Latin musical rhythms, Latin dance and it is usually practice in groups, would facilitates the intrinsic motivation. This could be the reason why previous Zumba^®^ intervention studies indicated that the adherence of the exercise program was high [47,48].

Some limitations need to be presented in this systematic review and meta-analysis. The first one is related to search strategy and study selection criteria, because only articles published in English language were included. A few databases were used, and it can represent another limitation. Moreover, some studies were not included because they did not report the required information for the meta-analysis, which means that the sample size is reduced. In addition, the majority of participants included in this systematic review and meta-analysis were female. In this regard, previous studies pointed that females had lower VO_2peak_ and higher fat mass than males. Thus, the results of this meta-analysis should be extrapolated to males with caution. Therefore, all these limitations need to be considered with respect to the findings of this study.

Therefore, future studies that analyze the effect of Zumba^®^ practice or interventions in different populations could help to clarify and highlight the potential benefits that may have this type of physical activities for health and fitness status in different populations. Likewise, it could be interesting to conduct future researches that explore the effects of Zumba^®^ practices in combination with other modalities of physical activities or training regimes or analyze the effect on body composition and VO_2peak_ of Zumba^®^ practices in contrast with other training programs or alternative physical activities.

## 5. Conclusions

The current evidence demonstrates the benefits of Zumba^®^ aerobic dance interventions to improve VO_2peak_, but not total fat mass (%) in adults. Based on the scarce scientific literature, clinical recommendations cannot be provided, and more studies are needed to estimate the real effects of Zumba^®^ practice on body composition as well as on VO_2peak_.

## Figures and Tables

**Table 1:** Characteristics of the sample.

RCT	Weeks	Sample Size of Groups and Sex	Age
Delextrat, Warner, Graham, and Neupert (2016)	8	ZU: 22 (females) CG: 22 (females)	26.6 (5.4) 27.9 (6.0)
Barene, Krustrup, Brekke, and Holtermann (2014)	40	ZU: 25 (0 males, 25 females) CG: 25 (2 males, 23 females)	45.9 (9.6) 47.4 (9.5)
Barene, Krustrup, Jackman, Brekke, and Holtermann (2013)	12	ZU: 35 (0 males, 30 females) CG: 35 (3 males, 31 females)	45.9 (9.6) 47.4 (9.5)
Barranco-Ruiz (2019)	16	ZU: 39 (females) CG: 31 (females)	39.87 (7) 38.19 (5.6)
Erzeybek (2020)	8	ZU: 9 (females) CG: 9 (females)	26.5 (1.8) 28.3 (3.2)
Gucluover (2020)	8	ZU: 14 (females) CG: 14 (females)	18–35
Guerendiain (2018)	16	ZU: 38 (86.84% females) CG: 28 (71.43% females)	38.24 (8.09 39.32 (6.40)
Muhammad (2019)	6	ZU: 29 (females) CG: 28 (females)	21.1 (0.4) 21.4 (0.4)
Barranco-Ruiz, and Villa-González (2020)	16	ZU: 33 (females) CG: 33 (females)	38.06 (7.11) 38.06 (7.11)
Domene, Moir, Pummell, Knox, and Easton (2016)	8	ZU: 10 (females) CG: 10 (females)	33 (11) 35 (13)

RCT: Randomized Controlled Trial; ZU: Zumba^®^ group; CG: Control group.

**Table 2:** Characteristics of the interventions.

RCT	Exercise Group	Control Group	Duration	Days Per	Training
	Type of Exercise	Type of Exercise		Week	Sessions
Delextrat, Warner, Graham, and Neupert (2016) [26]	Zumba^®^ workouts (1-h length each one)	Carried on normal daily activities	8 weeks	3	24
	Classes performed at home following a DVD				
Barene, Krustrup, Brekke, and Holtermann (2014) [28]	Zumba^®^ workouts (1-h length)	No intervention	40 weeks	2–3	NR
	Certified instructor				
Barene, Krustrup, Jackman, Brekke, and Holtermann (2013) [27]	Zumba^®^ workouts (1-h length)	No intervention	12 weeks	2–3	NR
	Certified instructor				
Barranco-Ruiz (2019) [33]	Zumba^®^ workouts (1-h length) Certified instructor	No intervention	16 weeks	3	NR
Erzeybek (2020) [31]	Zumba^®^ workouts (45-min length)	Oscillation training	8 weeks	3	NR
Gucluover (2020) [30]	Zumba^®^ workouts (1-h length)	Carried on normal daily activities	8 weeks	3	NR
Guerendiain (2018) [29]	Zumba^®^ workouts (1-h length)	Zumba^®^ workouts plus body weight	16 weeks	3	NR
Muhammad (2019) [32]	Zumba^®^ workouts (1-h length)	Educational program	6 weeks	2	NR
Barranco-Ruiz, and Villa-González (2020) [34]	Zumba^®^ workouts (1-h length) Certified instructor	No intervention	16 weeks	3	NR
Domene, Moir, Pummell, Knox, and Easton (2016) [21]	Zumba^®^ workouts (1-h length each one) classes were taught by certified instructor	Carried on normal daily activities	8 weeks	1–2	12

RCT: Randomized Controlled Trial; NR: not reported.

**Table 3:** Effects of interventions on total fat mass (%).

Authors	Weeks	Groups	Pre-Test			Post-Test			Change (Δ)	*P*-Values	
										Intragroup	Between Groups
Delextrat, Warner, Graham, and Neupert (2016) [26]	8	ZU	26.7	±	5.7	26.4	±	5.1	−0.3	NS	0.908
		CG	23.5	±	5.7	23.6	±	3.5	0.1	NS	
Barene, Krustrup, Brekke, and Holtermann (2013) [27]	12	ZU	35.9	±	5.8	34.9	±	5.7	−1	-	0.07
		CG	36.3	±	6.4	35.9	±	6.4	−0.4	-	
Barene, Krustrup, Jackman, Brekke, and Holtermann (2014) [28]	40	ZU	35.9	±	5.8	35.2	±	5.8	−0.7	-	0.003
		CG	36.3	±	6.4	36.9	±	6.6	0.6	-	
Barranco-Ruiz (2019) [33]	16	ZU	22.74	±	4.31	19.75	±	4.22	−2.99	S	<0.001
		CG	19.95	±	1.88	20.31	±	3.54	−0.36	NS	
Erzeybek et al. (2020) [31]	8	ZU	22.74	±	2.17	21.84	±	7.42	−0.9	NS	0.157
		CG	29.09	±	5.51	27.03	±	5.73	−2.06	S	
Gucluover (2020) [30]	8	ZU	31.66	±	7.34	30.22	±	7.16	−1.44	S	NR
		CG	30.78	±	8.38	31.40	±	8.41	0.62	NS	
Guerendiain (2018) [29]	6	ZU	22.13	±	4.48	18.80	±	3.40	−3.33	S	NS
		CG	21.36	±	3.08	17.99	±	2.91	−3.37	S	
Muhammad (2019) [32]	6	ZU	33.4	±	0.5	33	±	0.5	−0.4	NS	0.023
		CG	33.3	±	0.4	33.6	±	0.5	0.3	S	
Domene et al. (2016) [21]	8	ZU	30.9	±	5.5	-		-	−1.2	S	<0.05
		CG	31.7	±	5.8	-		-	0	NS	

ZG: Zumba^®^ group; CG: Control group; NS: Non-significant; NR: not reported.

**Table 4:** Effects of interventions on VO_2peak_ (mL kg^−1^ min^−1^).

Authors	Weeks	Groups	Pre-Test			Post-Test			Change (Δ)	*P*-Values	
										Intragroup	Between Groups
Domene, Moir, Pummell, Knox, and Easton (2016) [21]	8	ZU	29.4	±	5.9	NR	±	NR	3.1	S	<0.05
		CG	28.0	±	6.7	NR	±	NR	−0.7	NS	
Delextrat, Warner, Graham, and Neupert (2016) [26]	8	ZU	38.1	±	4.6	39.3	±	4.0	1.2	S	
		CG	38.3	±	5.3	37.3	±	5.4	−1.0	NS	0.01
Barene, Krustrup, Brekke, and Holtermann (2014) [28]	40	ZU	31.8	±	6.7	32.4	±	5.8	0.6	NR	0.001
		CG	33.1	±	6.7	33.4	±	6.9	0.3	NR	
Barene, Krustrup, Jackman, Brekke, and Holtermann (2013) [27]	12	ZU	31.8	±	6.7	32.8	±	6.3	1	NR	<0.05
		CG	33.1	±	6.7	32.6	±	6.8	−0.5	NR	
Barranco-Ruiz, and Villa-González (2020) [34]	16	ZU	34.67	±	3.94	36.00	±	3.84	1.33	S	0.030
		CG	32.63	±	5.87	32.49	±	6.27	−0.14	NS	

ZG: Zumba^®^ group; CG: Control group; NS: Non-significant; NR: not reported.

## Supplementary Materials

The following are available online at https://www.mdpi.com/1660-4601/18/1/105/s1.
