# Supplementary material for: Zumba®, Fat Mass and Maximum Oxygen Consumption: A Systematic Review and Meta-Analysis
Source: Int J Environ Res Public Health. 2020 Dec 25;18(1):105. doi: 10.3390/ijerph18010105 (PMC7794937; doi:10.3390/ijerph18010105)
Supplement: Supplementary file 1 [file ijerph-18-00105-s001.pdf]

Search strategy:

- **Pubmed**

For total fat mass (%):

("zumba") AND ("Waist circumference" OR "waist-hip" OR "fat" OR "weight" OR "BMI" OR "body composition" OR "body mass")

For $VO2_{peak}$:

("zumba") AND ("vo2" OR "oxygen" OR "VO2max").

- **Web of Science**

For total fat mass (%):

TS=("zumba") AND TS=("Waist circumference" OR "waist-hip" OR "fat" OR "weight" OR "BMI" OR "body composition" OR "body mass")

For $VO2_{peak}$:

TS=("zumba") AND TS=("vo2" OR "oxygen" OR "VO2max").
